# Evaluation of the Effect of Auxiliary Starter Yeasts With Enzyme Activities on Kazak Cheese Quality and Flavor

**DOI:** 10.3389/fmicb.2020.614208

**Published:** 2020-12-16

**Authors:** Jing Xiao, Yu Chen, Jie Li, Xuewei Shi, Li Deng, Bin Wang

**Affiliations:** ^1^College of Information Science and Technology, Shihezi University, Shihezi, China; ^2^Food College, Shihezi University, Shihezi, China

**Keywords:** Kazak cheese, yeasts, physicochemical indicators, volatile compounds, flavor

## Abstract

To investigate the effect of yeasts on Kazak cheese quality and flavor, three isolated yeasts (*Kluyveromyces marxianus* A2, *Pichia kudriavzevii* A11, and *Pichia fermentans* A19) were used to ferment cheeses and designated as StC, LhC, and WcC, respectively. The cheese fermented with a commercial lactic acid starter without adding yeast was used as control named LrC. The results showed that the texture of cheese added with yeasts were more brittle. *K. marxianus* A2 contributed to the formation of free amino acids and organic acids, especially glutamate and lactic acid. Moreover, *K. marxianus* A2 provides cheese with onion, oily, and floral aromas. Furthermore, *P. kudriavzevii* A11 promotes a strong brandy, herbaceous, and onion flavor. Although no significant aroma change was observed in PfC, it promoted the production of acetic acid, isoamyl acetate, and phenethyl acetate. These results indicate that yeasts are important auxiliary starters for cheese production.

## Introduction

Xinjiang is a multiethnic region in China. The Kazakhs, one of the primary minority nationalities in Xinjiang of China, produce Kazak cheeses with a unique craftsmanship (Zheng et al., 2018b). Kazak cheese is prepared from raw cow’s milk without adding an exogenous lactic acid bacteria (LAB) starter during the traditional production process, which can be identified with several stages: boiling, milk fermentation in goatskin bags, dehydration, shaping, and after-ripening (Zheng et al., 2018b, 2020). Furthermore, milk is believed to contain a large number of microorganisms, such as *Lactococcus*, *Staphylococcus*, *Corynebacterium*, *Brevibacterium*, *Clostridiisalibacter*, *Saccharomyces*, *Trichosporon*, and *Kluyveromyces* (Sun et al., 2014; Ryssel et al., 2015). The process with diverse and dynamic conditions led to the diversity of microorganism types. Some microorganisms with probiotic characteristics are preserved in ripened Kazak cheese (Zheng et al., 2018a). In the Kazakh diet, cheeses act as an excellent carrier for viable probiotic microorganisms and provide high levels of vitamins, calcium, oligosaccharides, and iron, compared with yogurt, milk powder, and condensed milk.

Lactic acid bacteria, such as *Lactobacillus* and *Lactococcus*, are identified as the main bacterial genera in the cheese fermentation process (Gao et al., 2017). During Kazak cheese making, milk is spontaneously fermented in a goatskin bag at room temperature (approximately 30°C) for 15 days, producing a high concentration of lactic acid (Zheng et al., 2018b). The increased lactic acid promotes a low pH value. The casein micelles demineralize to coagulate when the pH reaches the isoelectric point of casein (Fox et al., 2017; Kamimura et al., 2019). Recent studies have shown that except for LAB, a large number of yeasts also play an important role in cheese fermentation (Yuvaşen et al., 2018), such as *Pichia kudriavzevii*, *Kluyveromyces marxianus*, and *K. lactis* in Kazak artisanal cheese (Zheng et al., 2018a); *Yarrowia lipolytica*, *Debaryomyces hansenii*, and *P. fermentans* in artisanal short-ripened Galician cheeses (Atanassova et al., 2016); and *Torulaspora delbrueckii* and *Saccharomyces cerevisiae* in traditional Serbian cheeses (Zivkovic et al., 2015). It is believed that the LAB that become dominant during cheese fermentation originate from milk (Paxson, 2008), whereas yeasts are primarily derived from the storage environment and the fermentation process (Gonçalves Dos Santos et al., 2017). One study showed that *P. kudriavzevii*, *K. marxianus*, and *K. lactis*, which could provide a unique flavor to Kazak cheese, may originate from the environment, such as the milking, curd, storage, and ripening environment (Zheng et al., 2018b).

Yeasts exist in various fermented food because of their ability to adapt to low pH, high salt concentration, and low temperature storage conditions during milk fermentation (Chaveslopez et al., 2012). Besides, some yeasts can inhibit the growth of some enteric pathogens. For example, *K. lactis*, and *K. marxianus*, isolated from the Tomme d’orchie French cheese are able to inhibit the growth of *Listeria monocytogenes*, *Candida albicans*, and some *Bacillus* spp. (Ceugniez et al., 2015). During cheese fermentation, some yeasts, such as *D. hansenii*, *S. cerevisiae*, and *Y. lipolytica*, are beneficial to enhance the nutrition of cheese by releasing proteases, lipases, or β-galactosidases to convert proteins, lipids, and lactose of milk into small molecules (amino acids, fatty acids, and organic acids; Akpınar et al., 2011; Golić et al., 2013; Cardoso et al., 2015). Furthermore, *Y. lipolytica*, *K. lactis*, and *D. hansenii* also contribute to the formation of the texture and unique flavor of cheese by producing high concentrations of methyl ketone, butanoic acid, branched chain aldehydes, acetaldehyde, ethanol, and acetic acid esters (Atanassova et al., 2016; Juan et al., 2016). *K. lactis* also produce high levels of acetaldehyde, ethanol, branched chain aldehydes and alcohols, and acetic acid esters, which are the primary components of aroma and flavor in acid curd Cebreiro cheese (Atanassova et al., 2016).

Currently, flavors (Bergamaschi and Bittante, 2018), microorganisms (Yunita and Dodd, 2018; Yuvaşen et al., 2018), and their relationships (Bezerra et al., 2017; van Mastrigt et al., 2018) in cheese have become a research hotspot due to cheese providing excellent nutritional value with high digestibility and low allergenic potential (Bezerra et al., 2017). However, there is little research focusing on contribution of yeast to cheese flavors. In this study, to investigate the contribution of yeast to cheese flavor and quality, three yeasts, isolated from traditional Kazak cheese with protease, lipase, or β-galactosidase activity, were used to ferment cheese through co-fermentation with a commercial LAB starter. The physicochemical characteristics, free amino acids (FAAs), organic acids, texture, and volatile aromatic compounds in the ripened cheeses were investigated. The results will lay the theoretical foundation for the improvement of cheese flavor.

## Materials and Methods

### Isolation and Identification of Yeasts in Kazak Cheese

A total of 28 mature Kazak cheese samples were collected from local ethnic minority farmhouses in Xinjiang: Mulei regions (5 cheese), Yili (5), Altay (5), Balikun (5), and Tacheng (8) and stored at 4°C before the separation operation. The isolation method of yeasts was conducted as described by Zheng et al. (2018b) with some modification. First, 5 *g* cheese and 50 ml of sterile 0.9% sodium chloride solution were mixed and then homogenized in a rotary shaker (ZWYR-C2402; Shanghai Zhicheng Co., Ltd., Shanghai, China) for 30 min at 28°C. After being serially diluted, 100 μl of the corresponding dilutions was added to YPD (1% yeast extract, 2% peptone, and 2% glucose, 2% agar) agar-solidified medium (in triplicate) and then placed in a 28°C incubator (LRH-70; Shanghai Yiheng Technology Co., Ltd., Shanghai, China) to cultivate for 24 h. Single colonies were purified and stored in YPD broth with 30% glycerol at −20°C. Strains were identified by comparing sequences in the GenBank database^1^.

### Screening of Yeasts With Protease, Lipase, or β-Galactosidase Activities

According to the method described by Ozturkoglu-Budak et al. (2016), strains with protease activity can produce opaque hydrolyzed circles on YPD agar medium containing 2% skim milk. Quantitative measurements of protease activity were based on the method described by Mageswari et al. (2017) with modifications. First, 100 μl cell-free supernatants were mixed with 100 μl of 1% casein, which was dissolved in a 50 mM borax–NaOH buffer solution (pH 10) and incubated at 40°C for 10 min. The reaction was stopped by adding 200 μl of 0.4 M trichloroacetic acid. A blank control sample was also prepared by adding trichloroacetic acid before adding the enzyme solution. The test and blank solution were then centrifuged at 8,000 rpm for 5 min, after which 100 μl of filtrate was mixed with 500 μl 0.4 M Na_2_CO_3_ solution and 100 μl Folin–Ciocalteu reagent, and incubated at 40°C for 20 min. Finally, the absorbance was measured at a wavelength of 680 nm, and the enzymatic activity was calculated based on a tyrosine standard curve.

Strains with lipase activity were screened by adding 10% tributyrin (Yuanye Biotechnology Co., Ltd., Shanghai, China) to YPD, where a transparent circle will form around colonies with lipase activity. The method described by Konkit and Kim (2016) was used to assess lipase activity with some adjustments. First, 4 ml 12% olive oil and 5 ml 25 mM phosphate buffer solution (pH 7.0) were added to an Erlenmeyer flask and incubated at 40°C for 10 min. Then, 1.0 ml enzyme solution was added to the above mixture followed by an incubation at 37°C for 15 min. Finally, 15 ml 95% ethanol was added to the above mixture, which was then thoroughly mixed. Lipase activity was determined by titration with 50 mM NaOH after adding 3 drops of phenolphthalein to each sample and blank solutions.

Strains with β-galactosidase activity were screened by adding 20 mg/ml 5-bromo-4-chloro-3-indolyl-β-D-galactopyranoside (Sangon Biological Technology Co., Ltd., Shanghai, China) to YPD agar plates, where colonies testing positive for β-galactosidase activity turned blue. Measurement of β-galactosidase activity was based on the method of Souza et al. (2018). Furthermore, 50 μl enzyme solution was added to 50 μl 20 mM *o*-nitrophenyl-β-D-galactopyranoside substrate solution (Sangon Biological Technology), and the mixture was then incubated for 10 min at 37°C in a water bath (HH-1 Digital Thermostatic Water Bath; Junteng Electronic Instrument Co., Ltd., Shandong, China). The reaction was stopped by adding 200 μl 0.5 M sodium carbonate solution. Phosphate buffer without the enzyme solution was used as a blank control. The absorbance of the solutions was measured at 420 nm in a microplate reader (UV-1750; Shimadzu Corporation, Kyoto, Japan).

### Cheese Making and Sampling

A total of 120 L raw cow’s milk (Garden Dairy Co., Ltd., Xinjiang, China) was pasteurized at 75°C for 15 s and divided into four 30-L vats to make four different cheeses after cooled to 35°C. All the batches were first mixed with 1 × 10^6^ cfu/ml commercial LAB starter (Ci Enkang Biotechnology Co., Ltd., Jiangsu, China) for acidification. Then, *K. marxianus* A2 (file number in the GenBank database: MN985331.1), *P. fermentans* A19 (MN985333.1), and *P. kudriavzevii* A11 (MN985332.1) were added at a cell concentration of approximately 1 × 10^6^ cfu/ml to co-ferment with the commercial LAB starter. Before being added to the batches, the three yeasts were activated in a sterile medium consisting of 12% reconstituted skim milk, then incubated at 28°C for pre-ripening with 48 h. A batch without added yeast was used as a control. Then, all the batches were stirred for 10 min at 35°C and curdled at this temperature for 2 h. The curd was cut into 5-mm pieces to discharge the whey, and then was filtered with gauze and pressed in a mold to further remove the whey. Finally, the cheese was immersed in a 2% (*w*/*v*) salt solution for 1 h, then cut into small pieces and dried at 25°C for 2 days. All the cheese samples were stored in sterile bags under the same conditions (25°C, 65 ± 1% relative humidity).

For physicochemical and microbiological analysis, the cheeses were removed aseptically, transferred to sterile bags, and stored under the same conditions (25°C, 65 ± 1% relative humidity). Volatile compound analysis was performed at 10-day intervals for 40 days, and the cheese samples were frozen at −80°C and wrapped in vacuum plastic pouches.

### Physicochemical and Microbiological Analysis

The moisture of cheese was measured by direct drying in a laboratory oven at 105°C according to the Chinese national standard GB 5009.3-2016. Total protein was determined via the Kjeldahl method (follow the Chinese national standards GB 5009.5-2016) with Kjeldahl instruments (KjeIMaster K-375; BÜCHI Labortechnik AG, Switzerland). The determination of salt was performed with reference to the national standard GB 5009.42-2016. The pH was measured with a calibrated electronic digital pH meter (PHS-3C, Shanghai, China). The fat contents were determined by Soxhlet extraction following the Chinese national standard GB 5009.6-2016. Acidity was determined by the titration method according to the Chinese national standard GB 5413.34-2010. All samples were analyzed in triplicate.

Microbiological analysis was performed as Atanassova described with plate count method (Atanassova et al., 2016). Enumeration of the total viable microorganisms was performed on agar plates prepared with toxoid. LAB were cultured on MRS agar plates (peptone 10 g/L, beef powder 10 g/L, yeast extract 5 g/L, glucose 20 g/L, dipotassium hydrogen phosphate 2 g/L, diammonium hydrogen citrate 2 g/L, sodium acetate 5 g/L, magnesium sulfate 0.2 g/L, manganese sulfate 0.04 g/L, agar 14 g/L, and Tween 80 1 ml) containing natamycin and incubated at 35°C for 48 h under anaerobic conditions. Yeasts were enumerated on YPD medium containing 100 mg of chloramphenicol and after incubating at 28°C for 48 h.

### Determination of Free Amino Acids

The FAA levels in cheese samples were measured according to the method of Zhou et al. (2018). First, 2 *g* cheeses and 10 ml 6 M hydrochloric acid solution were transferred to a hydrolysis tube, evacuated, sealed, and then hydrolyzed at 110°C for 24 h. The hydrolysate was filtered into a volumetric flask and brought to the appropriate volume with distilled water. Then, 2 ml of the hydrolysate was dried, dissolved with 2 ml sodium citrate buffer solution (pH 2.2), and then filtered through a 0.22-μm filter for analysis. The FAA contents were determined using a fully automatic amino acid analyzer (LBA800; Tianjin Rambo Co., Ltd., Tianjin, China; Niro et al., 2017). The total FAAs were identified and quantified based on the retention times and peak areas of a standard FAA mixture (Yuanye Biotechnology).

### Determination of Organic Acids

To pretreat the sample according to Murtaza et al. (2017), 2 *g* cheese was mixed with 10 ml water and centrifuged. Then, the supernatant was transferred to a 50-ml volumetric flask and the cheese precipitate was repeatedly extracted to the same volumetric flask. After being brought to a constant volume with ethanol, 10 ml of the above solutions was transferred to a distillation flask and rotated at 80°C to dryness, with a second drying step performed after adding 5 ml of ethanol. Finally, the dried sample residue was dissolved in 1 ml phosphoric acid solution, then filtered through a 0.22-μm filter and assayed via HPLC.

Organic acids were determined according to Belguesmia et al. (2014). A high-performance liquid chromatography instrument (HPLC, Shimadzu LC-2010, Tokyo, Japan) equipped with a 5-μm 250 × 4.6 mm Spursil C18 (LC) column (Dima Technology Co., Ltd., Guangzhou, China.) was used. The UV detector was set at 210 nm and the column oven at 40°C. The mobile phase was methanol and 0.1% phosphoric acid, and the isocratic elution was performed at a volume ratio of 3:97 with a flow rate of 0.7 ml/min. The sample volume injected was 20 μl. Quantification of organic acid contents in the samples was carried out by generating calibration curves of external standards, including tartaric acid, lactic acid, succinic acid, malic acid, and citric acid (Yuanye Biotechnology Co., Ltd., Shanghai, China).

### Determination of Texture

Texture properties were measured using a TA texture analyzer (TA.XTPlus; Stable Micro System Co., United Kingdom) and a P/36R cylindrical probe (TA15/1000, 458 Å, 36 mm diameter) by reference to the method of Bekele et al. (2019). The test mode was TPA, the compression rate was 5 mm/s, the test speed was 1 mm/s, the speed after test was 5 mm/s, the trigger force was 5 *g*, the interval between measurements was 5 s, the deformation of the samples was 50%, and two consecutive determinations were performed for each sample. Before the test, the cheese was removed from the refrigerator at 4°C and equilibrated for 1 h at room temperature, and were then cut into 2-cm pieces for measurement. The calculation of texture properties was based on the method described by Rehman et al. (2018).

### Determination of Volatile Aromatic Compounds

The volatile profiles of the cheeses were determined by headspace-solid phase microextraction gas chromatography–mass spectrometry (HS-SPME/GC–MS) according to the method of Ocak et al. (2015). First, 2 *g* cracked cheese was added to a 15-ml headspace vial with a PTFE silicone lined magnetic cap and 1 μl 40 μg/kg 2-octanol was used as an internal control. Volatile aromatic compounds were extracted with an aging DVB/CAR/PDMS fiber (50/30 μm; Agilent Technologies, Palo Alto, CA, United States). The SPME fiber was first desorbed for 5 min in the injection port at 230°C before use to reduce the carryover from the previous analysis, after which the SPME needle was introduced into the septum in the vial cap, and the fibers were exposed to the headspace for 40 min at 40°C to extract volatile compounds (Li et al., 2020; Zheng et al., 2020).

Analyses were performed with a gas chromatography system (Agilent 7890B; Agilent Technologies) consisting of an HP Innowax column (60 m × 0.25 mm × 0.25 μm). The following oven temperature program was used: 50°C maintained for 3 min and then increased at a rate of 2°C/min to 100°C maintaining for 25 min, and finally an increase at 10°C/min to 230°C maintaining for 3 min. Helium was used as carrier gas at a flow rate of 1 ml/min, and the electron impact mode was set at 70 eV. Identification was based on comparing the retention time (RT) with those published in the National Institute of Standards and Technology (NIST, https://webbook.nist.gov) mass spectral library, where only compounds with a matching score ≥ 800 were retrieved and recorded (Panseri et al., 2014). Retention index (RI) of each compound was calculated with the retention time according to using a series of *n*-alkanes (C_8_–C_40_). Moreover, calculated RI was matched with a reference value according to NIST database (Panseri et al., 2014; Xu et al., 2019). The concentrations of the volatile compound were calculated with semi-quantitative method by multiplying the internal standard concentration and the ratio of the peak area of the volatile compound in the sample to the internal standard peak area, as shown in the following formula (Lugaz et al., 2005; Zheng et al., 2018b):

RCVC=(PAVC/PAIS)×CIS

RCVC: relative concentration of volatile compounds;
PAVC: peak area of volatile compounds;
PAIS: peak area of internal standard;
CIS: concentration of internal standard.

To evaluate which compounds were responsible for the aroma of Kazak cheeses, odor activity values (OAVs) were obtained by dividing the concentration of each compound by the respective odor threshold (in water) reported in the literature for the aroma analysis (Gemert, 2011; Li et al., 2020).

### Statistical Analysis

One-way ANOVA (*p* < 0.05) and Duncan’s tests were performed with SPSS version 22 (IBM Corp., Armonk, NY, United States) for physicochemical, amino acid, organic acid, texture, and volatile compound analysis. A heat map was generated to show the trends in flavors during the five storage periods in different cheeses following R version 3.5.3. The effects of different yeasts on the aroma profiles were evaluated by principal component analysis (PCA) using SIMCA 14.1 (Umetrics, Sweden).

## Results and Discussion

### Screening of Yeasts With Enzymatic Activity and Cheese Making

In this study, a total of 86 yeast strains were screened from the Kazak cheeses produced from different regions in Xinjiang, which could be classified as *K. lactis* (9 strains), *K. marxianus* (28 strains), *P. kudriavzevii* (21 strains), *T. delbrueckii* (3 strains), *Candida parapsilosis* (2 strains), *P. fermentans* (15 strains), *Lodderomyces elongisporus* (5 strains), and *Clavispora lusitaniae* (3 strains). Among these strains, *K. marxianus* and *P. kudriavzevii* were the dominant species, followed by *P. fermentans* (Supplementary Table 1). Only 16 yeasts exhibited high protease, lipase, or β-galactosidase activities. Based on the enzymatic activity results (Supplementary Figure 1), both *K. marxianus* A2 and *P. fermentans* A19 were observed to possess protease, β-galactosidase, and lipase activities, whereas *P. kudriavzevii* A11 had only protease and β-galactosidase activity (Supplementary Table 2). In addition, *K. marxianus* A2 had the highest protease activity (135 U/ml), *P. kudriavzevii* A11 possessed the highest β-galactosidase activity (375 U/ml), and *P. fermentans* A19 had the highest lipase activity (227 U/ml). Based on the dominant flora and enzymatic activities, *K. marxianus* A2, *P. kudriavzevii* A11, and *P. fermentans* A19 were selected as representative strains to manufacture cheeses, referred to hereafter as KmC, PkC, and PfC, respectively.

### Physicochemical and Microbiological Analysis of Kazak Cheese

The physicochemical characteristics of the Kazak cheeses are shown in Table 1. Also, the results showed that no differences in the protein, fat, and salt contents or pH values were observed between the four cheeses, whereas significant differences in moisture and acidity were observed. Among the four types of cheese, the moisture content of the control cheese was the lowest (26.55%), whereas those in PkC were the highest (29.48%). The observed moisture contents were consistent with the characteristics of Kazak cheese as a hard cheese, which is different from that of other cheeses (Cichosz et al., 2014; Cuffia et al., 2017). Salt acts as a preservative in cheese, inhibiting the growth of spoilage microorganisms and contributing to the flavor of the cheese (Guinee, 2004). In the four assayed cheeses, the average salt content was 1.9%, with almost no loss observed in this study. These results suggested that the physicochemical indicators of cheese primarily depend on the milk material and the LAB starter. The addition of the three types of yeasts had no significant differences on the physicochemical properties of the cheeses, including the protein, fat, and salt contents and pH value.

**TABLE 1:** Physicochemical parameters and microbial counts (log cfu/g) in four Kazak cheeses.

**Composition**	**Control**	**KmC**	**PkC**	**PfC**
Protein (%)	21.27 ± 1.82^a^	22.62 ± 1.90^a^	23.28 ± 1.93^a^	21.52 ± 1.86^a^
Moisture (%)	26.55 ± 1.08^b^	28.61 ± 1.03^ab^	29.48 ± 1.15^a^	29.15 ± 1.27^a^
Fat (%)	29.35 ± 2.53^a^	28.75 + 2.78^a^	29.21 ± 2.49^a^	28.97 ± 2.65^a^
Salt (%)	1.94 ± 0.07^a^	1.92 ± 0.06^a^	1.91 ± 0.05^a^	1.92 ± 0.08^a^
pH	4.98 ± 0.75^a^	5.48 ± 0.93^a^	5.25 ± 0.79^a^	4.95 ± 0.82^a^
Acidity (%)	1.25 ± 0.07^a^	1.13 ± 0.04^b^	0.96 ± 0.06^c^	0.92 ± 0.05^c^
Total viable counts	9.5 ± 0.05^a^	9.3 ± 0.04^c^	9.5 ± 0.03^a^	9.4 ± 0.02^b^
LABs	9.2 ± 0.02^a^	9.0 ± 0.05^c^	9.1 ± 0.03^b^	8.9 ± 0.03^d^
Yeasts	nd	2.8 ± 0.02^c^	3.1 ± 0.04^a^	2.9 ± 0.03^b^

*^1^Data are mean ± SD of three replicate analyses (*n* = 3). ^*a–c*^Means with different superscripts within the same row are significantly (*P* < 0.05) different. ^*nd*^Means not detected.*

The highest microbial counts (9.5 log cfu/g) and LAB (9.2 log cfu/g) were determined in the control cheeses (Table 1), whereas the total viable microbes counts (9.3 log cfu/g) and yeast counts (2.8 log cfu/g) determined in the KmC made with *K. marxianus* A2 were lower than those observed in the PkC and PfC samples after 40 days of ripening, respectively. The average number of viable yeasts (3.1 log cfu/g) observed in PkC was significantly higher than those in other cheeses. The results indicate that the yeasts survived during the cheese-making process as previously observed in raw-milk Tetilla cheese (Centeno et al., 2017).

### Free-Amino-Acid Analysis

In cheese, the abundances of yeasts, such as *Metschnikowia reukaufii*, *Y. lipolytica*, *Metschnikowia pulcherrima*, and *P. kudriavzevii* (Akpınar et al., 2011; Zheng et al., 2018b), have been shown affect the release of proteases, which was vital for the formation of FAAs from proteins.

**FIGURE 1:**
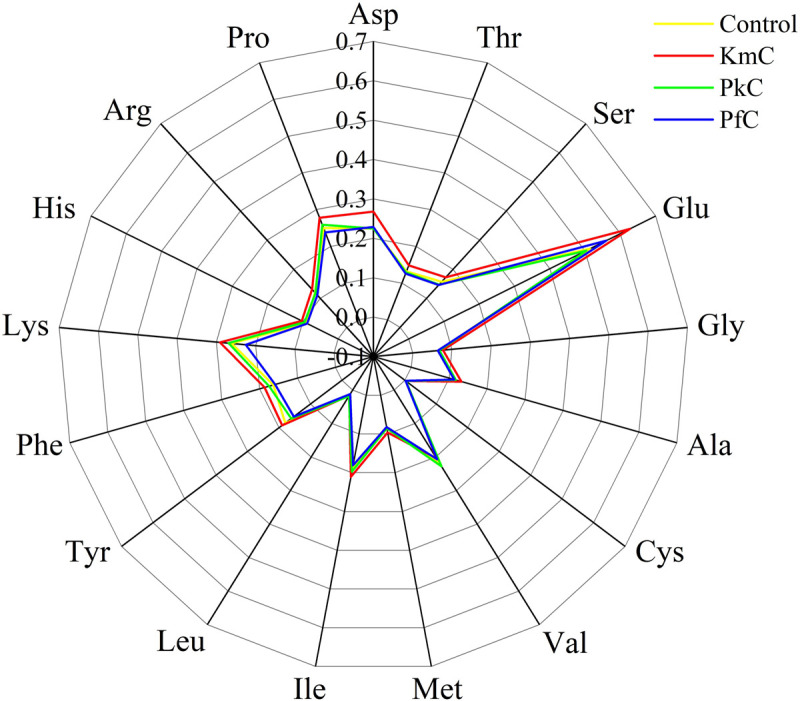
Amino acid contents in four cheeses. The letters in the rose wind direction figure indicate 17 kinds of amino acids.

In the four Kazak cheeses made in this study, the most abundant FAA was Glu, whereas the contents of Cys were the lowest (Figure 1). These results agree with previously reported data for Manchego cheese (Poveda et al., 2004), which was related to a number of synthetic pathways for Glu, such as the EMP pathway, HMP pathway, TCA cycle, and transamination reaction.

The total FAA contents in the four cheeses were different due to the addition of the yeasts used to ferment the cheese. In the KmC, a total FAA content of up to 3.13 *g*/100 *g* was observed, indicating that *K. marxianus* A2 contributes to the formation of FAAs (Supplementary Table 3). Furthermore, among the 17 amino acids measured, except for Val and Leu, the amino acid contents in the KmC were significantly higher than those observed in the other cheeses. This fact may be attributed to the high protease activity of *K. marxianus* A2. In contrast, the total FAA contents in the PfC were the lowest among the assayed cheeses (2.70 *g*/100 *g*). FAAs play an important role in the formation of cheese flavor, like *P. cactophila* and *K. lactis* were helpful to the production of 2-phenylethanol and isoamyl alcohol (Celinska et al., 2018). These results indicate that the addition of *K. marxianus* A2, which had the highest protease activity, resulted in significantly higher FAA contents than those observed in the other cheeses.

### Organic Acid Analysis

Organic acids are the primary source of acidic taste in foods (Lugaz et al., 2005; Da Conceicao Neta et al., 2007). The levels of tartaric acid, lactic acid, malic acid, succinic acid, and citric acid were investigated, and slight differences were observed in the contents of organic acids between the four cheese samples (Supplementary Table 4).

**FIGURE 2:**
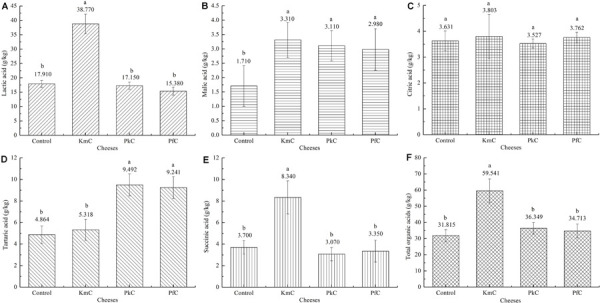
Organic acid contents in four cheeses. The letters control, KmC, PkC, and PfC represent cheeses fermented by a commercial lactic acid starter, a commercial lactic acid starter with *K. marxianus* A2, *P. fermentans* A19, and *P. Kudriavzevii* A11, respectively. **(A–F)** Represent lactic acid, malic acid, citric acid, tartaric acid, succinic acid and tatal organic acids, respectively. The letters in the column chart indicate whether the difference among data was statistically significant.

Among the several organic acids assayed, the concentration of lactic acid was significantly higher than that of the other organic acids, especially in the KmC with content up to 38.77 g/kg, whereas only 15.38 g/kg was detected in the PfC (Figure 2). A high lactic acid content would help lower the pH and subsequently affect the coagulation of casein. Besides, lactic acid also affects the flavor of cheese by altering the enzymatic activity and promoting the retention of coagulant in the curd (McSweeney, 2004). In addition, malic acid, citric acid, and succinic acid were also present at high levels in the KmC, while *P. kudriavzevii* A11 and *P. fermentans* A19 both promoted the generation of tartaric acid, the levels of which in the corresponding cheeses were as high as 9.49 and 9.24 *g*/kg, respectively. Because glycolytic and lipolytic activities greatly promote organic acids (Izco et al., 2002), as well as an important carbon source for microbial growth (Akalin et al., 2002), yeasts with enzymatic activity greatly promoted the production of organic acids through metabolism. Importantly, different organic acids produce different sour tastes. For example, citric acid provides a smooth sour taste and a fresh sensation, while malic acid provides a soft sour taste (Park et al., 2017).

The contents of malic acid and tartaric acids in the control cheese, which was only fermented by commercial LAB, were lower than those observed in the KmC, PkC, and PfC samples. Furthermore, the total organic acid contents in the KmC, PkC, and PfC samples (59.54, 36.35, and 34.71 g/kg) were also higher than that observed in the control. These results indicate that the addition of yeasts was beneficial to the formation of organic acids in cheese. Moreover, the differences of contents in organic acids depended largely on the yeasts used.

### Texture Analysis

**FIGURE 3:**
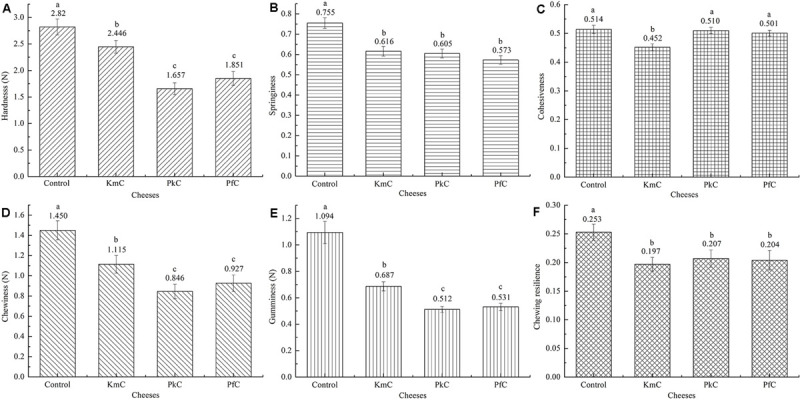
Texture analysis of four cheeses. The letters control, KmC, PkC, and PfC represent cheeses fermented by a commercial lactic acid starter, a commercial lactic acid starter with *K. marxianus* A2, *P. fermentans* A19, and *P. Kudriavzevii* A11, respectively. The letters in the column chart indicate whether the difference among data was statistically significant. **(A–F)** Represent hardness, springiness, cohesiveness, chewiness, gumminess and chewing resilience, respectively.

Analysis of the textural characteristics of the four cheeses showed that the hardness, springiness, cohesiveness, chewiness, gumminess, and chewing resilience were different (Figure 3).

All of the texture indicators, except for cohesiveness, in the KmC, PkC, and PfC samples were lower than those observed in the control, which may be related to acidification caused by the LAB starter (Supplementary Table 5). It is recognized that the pH, acidity, and moisture in food affect the cheese texture during the cheese fermentation process. However, a vital factor to the texture of cheeses is related to the proteolytic activity (Murtaza et al., 2014). The high contents of lactic acid promoted the hydration of casein, which further promotes the solubilization of proteins making the cheese less brittle (Dalié et al., 2010; Delavenne et al., 2012). The hardness, springiness, chewiness, and gumminess of the KmC were the highest among the cheeses assayed. These results indicate that the addition of yeasts led to a softer cheese texture compared with that of the control, which was highly related to the contents of lactic acid in the cheese.

### Volatile Compound Analysis

**FIGURE 4:**
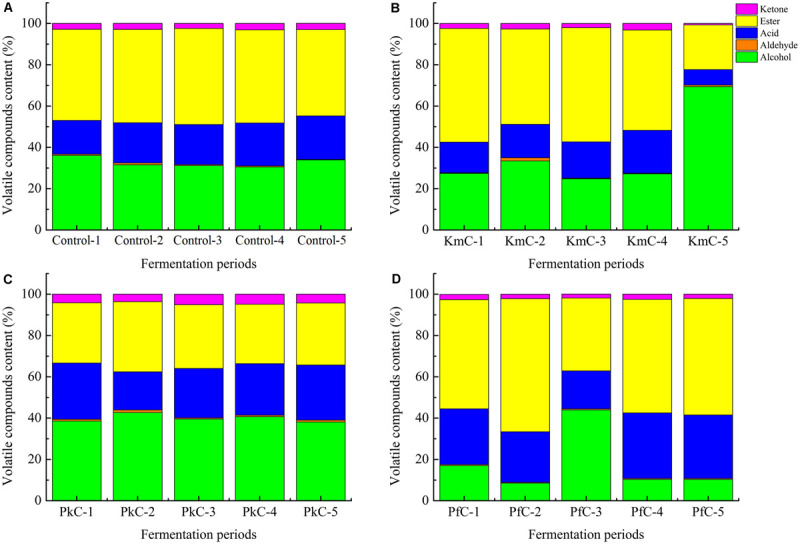
Classification of flavor compounds in Kazak cheese. The letters control **(A)**, KmC **(B)**, PkC **(C)**, and PfC **(D)** represent cheeses fermented by a commercial lactic acid starter, a commercial lactic acid starter with *K. marxianus* A2, *P. fermentans* A19, and *P. Kudriavzevii* A11, respectively. 1–5 represent 0, 10, 20, 30, and 40 days of fermentation time.

Fifty-five volatile compounds were detected in the cheeses, including 18 alcohols, 5 aldehydes, 11 acids, 17 esters, and 4 ketones (Table 2). Significant differences (*P* < 0.05) were observed for the amounts of flavor compounds between cheeses (Supplementary Figure 3). Among the four cheeses, alcohols and esters were the primary compound types, followed by acids (Figure 4), with large amounts of alcohols detected, such as ethanol, isoamylol, and 2,3-butanediol. Similarly, ethyl acetate, isoamyl acetate, and phenethyl acetate were abundant esters. These results were associated with alcohols and free fatty acids because of the reaction of esterification and alcoholysis that presented in cheeses (Bertuzzi et al., 2017). Acetic acid, isobutyric acid, butanoic acid, 2-methylcaproic acid, and hexanoic acid were the dominant acids of cheeses. Other volatile compounds such as methyl ketones were also detected. These volatiles can be produced by the esterification of an alcohol with a free carboxylic acid (Alewijn et al., 2005) or β-oxidation of fatty acids (Collins et al., 2003). As shown in Figure 4, we also observed that the level of esters, alcohols, and ketones were significantly different between the control cheese and PkC. However, in the KmC, the ester contents decreased, whereas that of alcohols gradually increased. In the PfC, the levels of alcohols tended to first increase and then decrease, whereas the changes in ester levels exhibited the opposite trend. The aforementioned results indicate that the addition of yeasts altered the composition and contents of volatile compounds, especially alcohols, esters, and acids.

**TABLE 2:** Concentrations of volatile compounds in Kazak cheeses sampled at 40 days (μg/kg).

**Compound**	**Calculated RI**	**IA**	**Control**	**KmC**	**PkC**	**PfC**
**Alcohols**						
Ethanol	932	MS	434.46 ± 9.6^b^	81.29 ± 2.5^d^	898.64 ± 17.8^a^	271.76 ± 7.5^c^
Isobutanol	1092	MS	24.36 ± 3.5^b^	21.73 ± 1.6^b^	50.92 ± 1.6^a^	9.38 ± 0.6^c^
Isoamylol	1209	MS	290.25 ± 7.3^b^	7.90 ± 0.7^d^	649.08 ± 12.4^a^	52.20 ± 1.2^c^
Pentanol	1250	MS, RI	4.37 ± 0.3^b^	92.30 ± 1.9^a^	5.97 ± 0.8^b^	4.69 ± 0.2^b^
4-Methyl-hexan-2-ol	1327	MS	1.16 ± 0.3^b^	195.04 ± 6.3^a^	4.97 ± 0.3^b^	2.46 ± 0.1^b^
1-Hexanol	1355	MS	5.19 ± 0.4^c^	14.40 ± 1.7^b^	20.94 ± 0.2^a^	5.97 ± 0.5^c^
3-Octanol	1393	MS, RI	1.87 ± 0.1^c^	40.22 ± 3.2^a^	5.75 ± 0.4^b^	0.90 ± 0.1^c^
2-Ethylhexanol	1491	MS, RI	1.27 ± 0.2^b^	1963.82 ± 35.7^a^	2.12 ± 0.1^b^	nd
2,3-Butanediol	1543	MS, RI	322.98 ± 1.8^c^	4071.46 ± 43.8^a^	416.49 ± 8.6^b^	117.34 ± 2.8^d^
1-Octanol	1557	MS, RI	3.62 ± 0.6^c^	7.62 ± 0.3^a^	6.48 ± 0.9^b^	3.55 ± 0.3^c^
Isobutoxypropanol	1582	MS	44.61 ± 1.3^b^	695.07 ± 38.2^a^	51.64 ± 1.5^b^	18.14 ± 0.9^b^
4-Methyl-2-pentenal	1604	MS	1.98 ± 0.1^b^	27.80 ± 1.7^a^	2.57 ± 0.2^b^	nd
1-Non-anol	1660	MS, RI	2.50 ± 0.2^bc^	11.57 ± 2.5^a^	4.13 ± 0.4^b^	1.52 ± 0.1^c^
1,3-Butanediol	1576	MS, RI	nd	1186.32 ± 32.6	nd	nd
Methionol	1719	MS, RI	2.58 ± 0.1^b^	1196.36 ± 28.5^a^	3.96 ± 0.2^b^	1.33 ± 0.3^b^
α-Cumyl alcohol	1773	MS, RI	0.41 ± 0.1^b^	6.49 ± 0.7^a^	nd	0.66 ± 0.1^b^
Phenylethanol	1906	MS, RI	128.63 ± 3.6^b^	222.00 ± 4.3^a^	1.23 ± 0.1^d^	9.71 ± 0.4^c^
2-Methylheptan-2-ol	1053	MS	Nd	8.75 ± 0.5	10.27 ± 0.9	nd
**Aldehydes**						
Hexanal	1083	MS, RI	0.64 ± 0.1^c^	36.69 ± 2.7^a^	30.32 ± 2.4^b^	2.13 ± 0.3^c^
2-Heptenal	1322	MS, RI	0.86 ± 0.1^c^	5.08 ± 0.4^a^	2.07 ± 0.3^b^	0.99 ± 0.1^c^
Non-anal	1391	MS, RI	5.94 ± 0.8^c^	58.71 ± 0.6^a^	15.24 ± 2.6^b^	13.93 ± 0.3^b^
Decanal	1498	MS, RI	nd	1.27 ± 0.2^b^	2.01 ± 0.2^a^	1.52 ± 0.1^b^
3-Butanolal	2423	MS	0.34 ± 0.1^c^	1.41 ± 0.1^a^	1.06 ± 0.1^b^	0.90 ± 0.1^b^
**Acids**						
Acetic acid	1449	MS, RI	465.28 ± 26.3^c^	187.14 ± 17.5^d^	815.79 ± 45.2^a^	728.22 ± 33.6^b^
Isobutyric acid	1570	MS, RI	98.11 ± 10.4^c^	56.73 ± 2.1^d^	195.13 ± 20.7^a^	167.41 ± 14.2^b^
Butanoic acid	1625	MS, RI	64.82 ± 9.4^d^	383.31 ± 21.8^a^	158.28 ± 17.3^b^	111.51 ± 7.7^c^
2-Methylcaproic acid	1757	MS, RI	55.18 ± 6.9^c^	8.47 ± 1.5^d^	159.84 ± 14.8^b^	285.36 ± 26.3^a^
Pentanoic acid	1733	MS, RI	0.90 ± 0.1^c^	6.49 ± 0.7^a^	1.84 ± 0.3^b^	1.61 ± 0.4^bc^
2-Methylvalerate	1764	MS	1.98 ± 0.2^b^	nd	1.73 ± 0.1^b^	5.87 ± 0.2^a^
Hexanoic acid	1846	MS, RI	69.60 ± 6.3^c^	nd	142.98 ± 7.3^a^	120.94 ± 5.7^b^
Heptanoic acid	1950	MS, RI	1.42 ± 0.2^b^	387.68 ± 37.4^a^	0.56 ± 0.1^b^	0.33 ± 0.1^b^
Non-anoic acid	2171	MS, RI	0.41 ± 0.1^b^	1.13 ± 0.1^b^	1.28 ± 0.2^b^	51.59 ± 2.5^a^
Octanoic acid	2060	MS, RI	25.07 ± 1.5^a^	16.09 ± 2.8^b^	0.17 ± 0.1^d^	8.15 ± 1.1^c^
Benzoic acid	2412	MS, RI	6.28 ± 1.8^b^	13.97 ± 1.5^a^	16.41 ± 2.1^a^	nd
**Esters**						
Ethyl acetate	888	MS, RI	620.10 ± 48.4^a^	493.96 ± 42.9^b^	690.90 ± 37.4^a^	267.28 ± 26.4^c^
Isobutyl acetate	1012	MS, RI	4.97 ± 1.1^c^	22.30 ± 1.6^b^	4.13 ± 1.3^c^	30.06 ± 2.9^a^
Ethyl butanoate	1035	MS, RI	12.59 ± 1.2^c^	178.53 ± 5.1^a^	30.65 ± 1.8^b^	nd
Isoamyl acetate	1122	MS, RI	571.08 ± 16.4^b^	36.69 ± 1.7^c^	573.21 ± 34.1^b^	1931.13 ± 41.7^a^
Ethyl hexanoate	1233	MS, RI	35.83 ± 2.9^b^	23.57 ± 2.8^c^	73.97 ± 6.3^a^	15.02 ± 2.8^d^
Hexyl acetate	1272	MS	2.32 ± 0.2^c^	10.02 ± 1.4^a^	nd	7.63 ± 1.2^b^
Ethyl tert-butylacetate	1334	MS	0.97 ± 0.1^b^	2.54 ± 0.6^a^	2.46 ± 0.2^a^	1.09 ± 0.1^b^
Ethyl propanoate	1365	MS	0.26 ± 0.1^b^	nd	3.07 ± 1.1^a^	0.85 ± 0.2^b^
Ethyl lactate	1427	MS	26.23 ± 5.2^b^	8.19 ± 0.03^d^	67.78 ± 2.1^a^	17.86 ± 3.2^c^
Ethyl caprylate	1435	MS, RI	55.70 ± 3.9^a^	39.94 ± 1.9^c^	47.34 ± 0.8^b^	25.01 ± 2.7^d^
Propyleneacetate	1526	MS, RI	1.91 ± 0.1^b^	147.06 ± 3.2^a^	4.91 ± 1.1^b^	1.75 ± 0.1^b^
Glycol diacetate	1535	MS, RI	4.03 ± 0.8^b^	45.02 ± 2.1^a^	4.30 ± 1.2^b^	1.23 ± 0.2^c^
Ethyl caprate	1638	MS, RI	3.44 ± 0.6^b^	4.80 ± 1.7^b^	3.91 ± 1.8^b^	15.54 ± 2.5^a^
Phenethyl acetate	1815	MS, RI	212.17 ± 24.7^c^	1750.72 ± 57.2^a^	154.59 ± 25.8^c^	319.56 ± 16.4^b^
Phenethyl butyrate	1958	MS, RI	2.88 ± 0.8^b^	208.45 ± 24.8^a^	1.40 ± 0.3^b^	0.28 ± 0.1^b^
D-(-)-Pantolactone	2029	MS, RI	0.49 ± 0.1^b^	18.21 ± 1.9^a^	0.50 ± 0.1^b^	nd
5-Decanolide	2194	MS, RI	1.46 ± 0.2^c^	7.34 ± 0.3^a^	nd	2.27 ± 0.2^b^
**Ketones**						
2-Heptanone	1078	MS, RI	13.97 ± 3.2^b^	14.54 ± 2.8^b^	49.30 ± 4.2^a^	15.40 ± 0.8^b^
Acetoin	1284	MS, RI	85.55 ± 9.4^b^	8.47 ± 1.9^d^	154.31 ± 5.8^a^	47.32 ± 6.3^c^
6-Methyl-5-hepten-2-one	1338	MS, RI	0.93 ± 0.1^c^	13.27 ± 2.6^a^	9.04 ± 0.3^b^	8.62 ± 1.4^b^
2-Non-anone	1390	MS, RI	12.18 ± 1.2^c^	54.90 ± 4.2^a^	27.41 ± 3.9^b^	28.94 ± 2.8^b^

*^1^Data are mean ± SD of three replicate analyses (*n* = 3). ^*a–d*^Means with different superscripts within the same row are significantly (*P* < 0.05) different. ^2^Control, KmC, PkC, and PfC represent samples collected on 40 days. ^*nd*^Means not detected. IA, identification approach.*

### Dynamic Changes in Volatile Compounds and Aroma Evaluation in Cheeses

**FIGURE 5:**
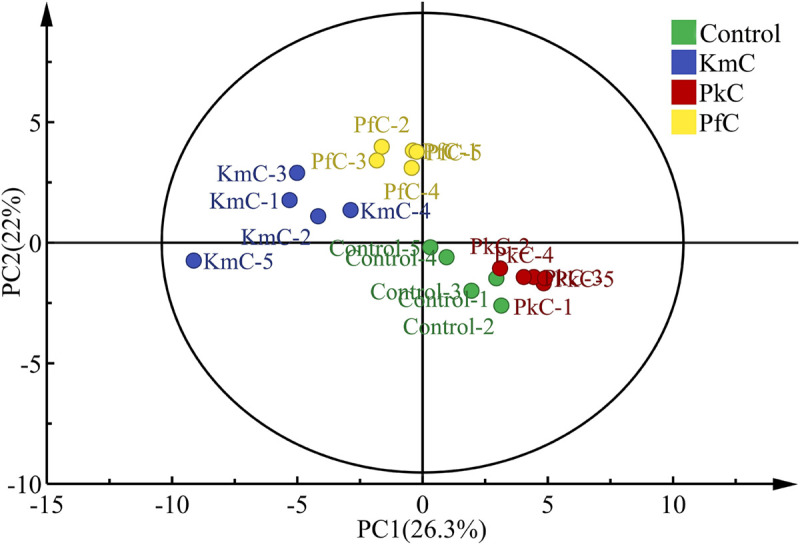
Principal component analysis of four Kazak cheeses according to the determined volatile compounds. The letters control, KmC, PkC, and PfC represent cheeses fermented by a commercial lactic acid starter, a commercial lactic acid starter with *K. marxianus* A2, *P. fermentans* A19, and *P. Kudriavzevii* A11, respectively. 1–5 represent 0, 10, 20, 30, and 40 days of fermentation time.

The aromatic profiles of the four Kazak cheeses on different days (0, 10, 20, 30, 40 days) were determined, and the relationships between the different yeast strains and the composition of the volatile compounds were analyzed via PCA. The results showed that PC1 and PC2 could explain 26.3 and 22% of the observed variance, respectively (Figure 5). The differences between selected yeasts and the volatile compounds produced could be easily distinguished by the concentrations of isoamylol (A3), 1-hexanol (A6), 2,3-butanediol (A9), methionol (A15), decanal (B4), 3-butanolal (B5), heptanoic acid (C8), ethyl acetate (D1), and ethyl hexanoate (D5; Supplementary Figure 2).

**FIGURE 6:**
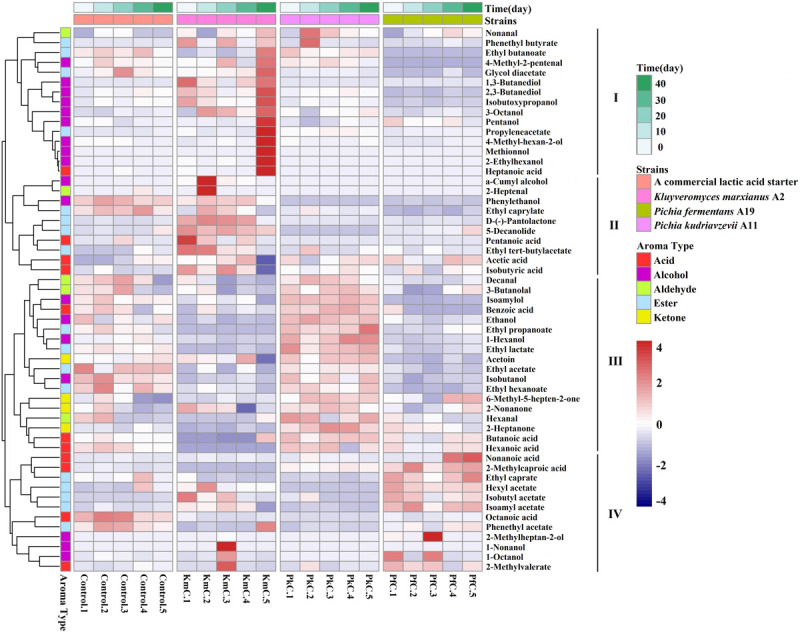
Correlation analysis between five fermentation periods of strains. The letters control, KmC, PkC, and PfC represent cheeses fermented by a commercial lactic acid starter, a commercial lactic acid starter with *K. marxianus* A2, *P. fermentans* A19, and *P. Kudriavzevii* A11, respectively. 1–5 represent 0, 10, 20, 30, and 40 days of fermentation time.

A heat map was used to compare the trends in the levels of 55 flavor compounds observed during the five ripening periods. According to the clustering results, these compounds could be divided into four categories (I, II, III, and IV) that contained 15, 10, 18, and 12 aromatic compounds, respectively (Figure 6). The results showed that not only the LAB contributed to the flavor of the cheese but also *K. marxianus* A2, *P. kudriavzevii* A11, and *P. fermentans* A19 strains were associated with proteolysis, lipolysis, or lactose degradation. *K. marxianus* A2 contributed to the formation of compounds in categories I and II, *P. kudriavzevii* A11 had an important effect on the compounds in category III, whereas *P. fermentans* A19 primarily promoted the contents of compounds in category IV. These results indicate that adding yeasts could alter the flavor of cheeses and that *K. marxianus* A2, *P. kudriavzevii* A11, and *P. fermentans* A19 were good producers of flavor compounds.

**FIGURE 7:**
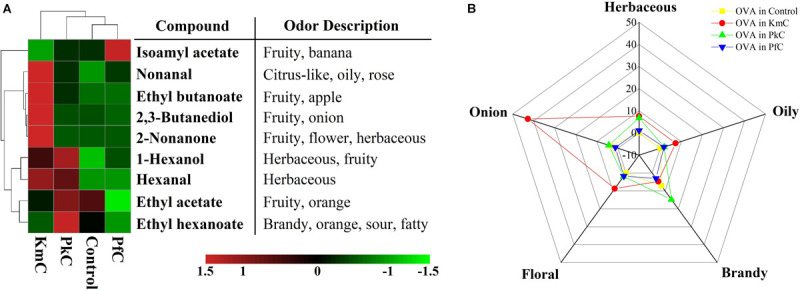
Aroma profile of four Kazak cheeses. **(A)** Odor description and odor activity value (OAV) of aroma compounds in Kazak cheese. **(B)** The letters control, KmC, PkC, and PfC represent cheeses fermented by a commercial lactic acid starter, a commercial lactic acid starter with *K. marxianus* A2, *P. fermentans* A19, and *P. Kudriavzevii* A11, respectively.

Yeast can effectively produce many secondary metabolites that are crucial to the quality of cheese, including carbonyl compounds, sulfur compounds, fatty acid derivatives, phenolic compounds, and higher alcohols, which have been directly related to the aroma of cheeses (Dzialo et al., 2017). Volatile compounds with OAV > 1 were divided into six aromas, comprising fruity, herbaceous, floral, fatty/oily, brandy, and onion. Volatile compounds in the KmC, PfC, and PkC samples exhibited a richer flavor than that of the control (Figure 7). Among the identified volatile compounds, the OAV of ethyl acetate was the highest, especially in the PkC, followed by the control, KmC, and PfC, which indicates that fruity aroma was abundant in the four cheeses. Furthermore, except for hexanal, all other volatile compounds detected possessed a specific fruity aroma. High OAVs were also observed for isoamyl acetate (64.37), which has a banana odor in PfC and 2,3-butanediol (42.81) possesses an onion odor quality in KmC (Supplementary Table 6). Furthermore, non-anal with oily, rose, and citrus-like notes, ethyl butanoate with an apple aroma, and ethyl hexanoate with brandy, orange, and sour odor notes in the KmC had OAVs of 7.34, 9.92, and 4.71, respectively. OAVs higher than one were also determined for 1-hexanol, hexanal, and 2-non-anone in the different cheeses. Evaluation of aroma characteristics other than fruity flavor showed that the cheese produced with *K. marxianus* A2 possessed a strong onion, oily, and floral aroma, whereas *P. kudriavzevii* A11 contributed to the formation of brandy, herbaceous, and onion flavors in the PkC, and no significant aroma changes were observed in PfC made with *P. fermentans* A19 (Figure 7B).

## Conclusion

The results of this study showed that yeasts were primary microbial components in Kazak cheese and contributed to cheese quality and flavors. In general, adding yeast had no effect on the physicochemical parameters like pH value or protein, fat, and salt contents of cheeses, except for moisture and acidity. The cheeses made with added yeasts were more brittle than that of control cheese. Furthermore, the yeast added in this study had complex and diverse effects on cheese flavors. *K. marxianus* A2 contributed to the formation of FAA and organic acids in cheese, especially Glu and lactic acid. Besides, this yeast also promoted the contents of 2-ethylhexanol, 2,3-butanediol, isobutoxypropanol, 1,3-butanediol, methionol, and phenethyl acetate, providing onion, oily, and floral aromas to the cheese. In contrast, *P. kudriavzevii* A11 contributed to the accumulation of ethanol, isoamylol, acetic acid, ethyl acetate, and isoamyl acetate, promoting a strong brandy, herbaceous, and onion flavor. Thus, the addition of yeasts increases the flavors and distinctiveness of Kazak cheese, indicating that yeasts are important auxiliary starters for cheese production.

## Data Availability Statement

The raw data supporting the conclusions of this article will be made available by the authors, without undue reservation.

## Author Contributions

YC did the experiment, collected test data, and drafted the article. JX and YC analyzed data and submitted amendments to the article. XS, LD, and JL conceived and designed the study. BW revised the article. All authors contributed to the article and approved the submitted version.

## Conflict of Interest

The authors declare that the research was conducted in the absence of any commercial or financial relationships that could be construed as a potential conflict of interest.

## Footnotes

1 https://blast.ncbi.nlm.nih.gov/blast.cgi

## References

[B1] Akalin A. S. Gonc S. Akbas Y. (2002). Variation in organic acids content during ripening of Pickled white cheese. *J. Dairy Sci.* 85 1670–1676. 10.3168/jds.S0022-0302(02)74239-2 12201516

[B2] Akpınar O. Uçar F. Yalçın H. T. (2011). Screening and regulation of alkaline extracellular protease and ribonuclease production of Yarrowia lipolytica strains isolated and identified from different cheeses in Turkey. *Ann. Microbiol.* 61 907–915. 10.1007/s13213-011-0213-x

[B3] Alewijn M. Sliwinski E. L. Wouters J. T. M. (2005). Production of fat-derived (flavour) compounds during the ripening of gouda cheese. *Int. Dairy J.* 15 733–740. 10.1016/j.idairyj.2004.09.009

[B4] Atanassova M. R. Fernández-Otero C. Rodríguez-Alonso P. Fernández-No I. C. Garabal J. I. Centeno J. A. (2016). Characterization of yeasts isolated from artisanal short-ripened cows’ cheeses produced in Galicia (NW Spain). *Food Microbiol.* 53 172–181. 10.1016/j.fm.2015.09.012 26678145

[B5] Bekele B. Hansen E. B. Eshetu M. Ipsen R. Hailu Y. (2019). Effect of starter cultures on properties of soft white cheese made from camel (*Camelus dromedarius*) milk. *J. Dairy Sci.* 102 1108–1115. 10.3168/jds.2018-15084 30591338

[B6] Belguesmia Y. Rabesona H. Mounier J. Pawtowsky A. Blay G. L. Barbier G. (2014). Characterization of antifungal organic acids produced by *Lactobacillus* harbinensis K.V9.3.1Np immobilized in gellan–xanthan beads during batch fermentation. *Food Control* 36 205–211. 10.1016/j.foodcont.2013.08.028

[B7] Bergamaschi M. Bittante G. (2018). From milk to cheese: evolution of flavor fingerprint of milk, cream, curd, whey, ricotta, scotta, and ripened cheese obtained during summer Alpine pasture. *J. Dairy Sci.* 101 3918–3934. 10.3168/jds.2017-13573 29454692

[B8] Bertuzzi A. S. Kilcawley K. N. Sheehan J. J. Osullivan M. G. Kennedy D. Mcsweeney P. L. H. (2017). Use of smear bacteria and yeasts to modify flavour and appearance of Cheddar cheese. *Int. Dairy J.* 72 44–54. 10.1016/j.idairyj.2017.04.001

[B9] Bezerra T. K. A. Arcanjo N. M. D. O. Garcia E. F. Gomes A. M. P. Queiroga R. D. C. R. D. E. De Souza E. L. (2017). Effect of supplementation with probiotic lactic acid bacteria, separately or combined, on acid and sugar production in goat ‘coalho’ cheese. *LWT* 75 710–718. 10.1016/j.lwt.2016.10.023

[B10] Cardoso V. M. Borelli B. M. Lara C. A. Soares M. A. Pataro C. Bodevan E. C. (2015). The influence of seasons and ripening time on yeast communities of a traditional Brazilian cheese. *Food Res. Int.* 69 331–340. 10.1016/j.foodres.2014.12.040

[B11] Celinska E. Bonikowski R. Bialas W. Dobrowolska A. Sloma B. Borkowska M. (2018). Pichia cactophila and kluyveromyces lactis are highly efficient microbial cell factories of natural amino acid-derived aroma compounds. *Molecules* 23:97. 10.3390/molecules23010097 29301324 PMC6017828

[B12] Centeno J. A. Garabal J. I. Docampo F. Lorenzo J. M. Carballo J. (2017). Recovering traditional raw-milk Tetilla cheese flavour and sensory attributes by using Kocuria varians and *Yarrowia* lipolytica adjunct cultures. *Int. J. Food Microbiol.* 251 33–40. 10.1016/j.ijfoodmicro.2017.03.014 28384620

[B13] Ceugniez A. Drider D. Jacques P. Coucheney F. (2015). Yeast diversity in a traditional French cheese “Tomme d’orchies” reveals infrequent and frequent species with associated benefits. *Food Microbiol.* 52 177–184. 10.1016/j.fm.2015.08.001 26338133

[B14] Chaveslopez C. Tofalo R. Serio A. Paparella A. Sacchetti G. Suzzi G. (2012). Yeasts from Colombian Kumis as source of peptides with Angiotensin I converting enzyme (ACE) inhibitory activity in milk. *Int. J. Food Microbiol.* 159 39–46. 10.1016/j.ijfoodmicro.2012.07.028 22938834

[B15] Cichosz G. Aljewicz M. Nalepa B. (2014). Viability of the *Lactobacillus rhamnosus* HN001 probiotic strain in Swiss- and Dutch-Type cheese and Cheese-Like products. *J. Food Sci.* 79 1181–1188. 10.1111/1750-3841.12458 24784351

[B16] Collins Y. F. Mcsweeney P. L. H. Wilkinson M. G. (2003). Evidence of a relationship between autolysis of starter bacteria and lipolysis in Cheddar cheese during ripening. *J. Dairy Res.* 70 105–113. 10.1017/S0022029902005915 12617399

[B17] Cuffia F. George G. Renzulli P. Reinheimer J. Meinardi C. A. Burns P. (2017). Technological challenges in the production of a probiotic pasta filata soft cheese. *LWT* 81 111–117. 10.1016/j.lwt.2017.03.039

[B18] Da Conceicao Neta E. R. Johanningsmeier S. D. Drake M. A. McFeeters R. F. (2007). A chemical basis for sour taste perception of acid solutions and fresh-pack dill pickles. *J. Food Sci.* 72 S352–S359. 10.1111/j.1750-3841.2007.00400.x 17995690

[B19] Dalié D. K. D. Deschamps A. M. Richard-Forget F. (2010). Lactic acid bacteria – Potential for control of mould growth and mycotoxins: a review. *Food Control* 21 370–380. 10.1016/j.foodcont.2009.07.011

[B20] Delavenne E. Mounier J. Déniel F. Barbier G. Le Blay G. (2012). Biodiversity of antifungal lactic acid bacteria isolated from raw milk samples from cow, ewe and goat over one-year period. *Int. J. Food Microbiol.* 155 185–190. 10.1016/j.ijfoodmicro.2012.02.003 22364725

[B21] Dzialo M. C. Park R. Steensels J. Lievens B. Verstrepen K. J. (2017). Physiology, ecology and industrial applications of aroma formation in yeast. *FEMS Microbiol. Rev.* 41(Suppl._1), S95–S128. 10.1093/femsre/fux031 28830094 PMC5916228

[B22] Fox P. F. Guinee T. P. Cogan T. M. McSweeney P. L. H. (2017). “Fresh cheese products: principals of manufacture and overview of different varieties^∗^,” in *Fundamentals of Cheese Science*, (Boston, MA: Springer), 543–588.

[B23] Gao M. L. Hou H. M. Teng X. X. Zhu Y. L. Hao H. S. Zhang G. L. (2017). Microbial diversity in raw milk and traditional fermented dairy products (Hurood cheese and Jueke) from Inner Mongolia. *China. Genet. Mol. Res.* 16:gmr16019451. 10.4238/gmr16019451 28290619

[B24] Gemert L. J. V. (2011). *Compilations of Odour Threshold Values in Air, Water and Other Media (second Enlarged and Revised Edition). No. 1.* Utrecht: Oliemans Punter & Partners.

[B25] Golić N. Čadež N. Terzić-Vidojević A. Šuranská H. Beganović J. Lozo J. (2013). Evaluation of lactic acid bacteria and yeast diversity in traditional white pickled and fresh soft cheeses from the mountain regions of Serbia and lowland regions of Croatia. *Int. J. Food Microbiol.* 166 294–300. 10.1016/j.ijfoodmicro.2013.05.032 23973841

[B26] Gonçalves Dos Santos M. T. P. Benito M. J. Córdoba M. D. G. Alvarenga N. Ruiz-Moyano Seco de Herrera S. (2017). Yeast community in traditional Portuguese Serpa cheese by culture-dependent and -independent DNA approaches. *Int. J. Food Microbiol.* 262 63–70. 10.1016/j.ijfoodmicro.2017.09.013 28964999

[B27] Guinee T. P. (2004). Salting and the role of salt in cheese. *Int. J. Dairy Technol.* 57 99–109. 10.1111/j.1471-0307.2004.00145.x

[B28] Izco J. M. Tormo M. Jiménez-Flores R. (2002). Rapid simultaneous determination of organic acids, free amino acids, and lactose in cheese by capillary electrophoresis. *J. Dairy Sci.* 85 2122–2129. 10.3168/jds.S0022-0302(02)74290-2 12362443

[B29] Juan B. Zamora A. Quevedo J. M. Trujillo A.-J. (2016). Proteolysis of cheese made from goat milk treated by ultra high pressure homogenisation. *LWT* 69 17–23. 10.1016/j.lwt.2015.12.013

[B30] Kamimura B. A. De Filippis F. Sant’Ana A. S. Ercolini D. (2019). Large-scale mapping of microbial diversity in artisanal Brazilian cheeses. *Food Microbiol.* 80 40–49. 10.1016/j.fm.2018.12.014 30704595

[B31] Konkit M. Kim W. (2016). Activities of amylase, proteinase, and lipase enzymes from Lactococcus chungangensis and its application in dairy products. *J. Dairy Sci.* 99 4999–5007. 10.3168/jds.2016-11002 27108177

[B32] Li J. Huang Q. Zheng X. Ge Z. Lin K. Zhang D. (2020). Investigation of the lactic acid bacteria in kazak cheese and their contributions to cheese fermentation. *Front. Microbiol.* 11:228. 10.3389/fmicb.2020.00228 32226414 PMC7080652

[B33] Lugaz O. Pillias A. M. Boireau-ducept N. Faurion A. (2005). Time-intensity evaluation of acid taste in subjects with saliva high flow and low flow rates for acids of various chemical properties. *Chem. Senses* 30 89–103. 10.1093/chemse/bji004 15647467

[B34] Mageswari A. Subramanian P. Chandrasekaran S. Karthikeyan S. Gothandam K. M. (2017). Systematic functional analysis and application of a cold-active serine protease from a novel Chryseobacterium sp. *Food Chem.* 217 18–27. 10.1016/j.foodchem.2016.08.064 27664603

[B35] McSweeney P. L. H. (2004). Biochemistry of cheese ripening. *Int. J. Dairy Technol.* 57 127–144. 10.1111/j.1471-0307.2004.00147.x

[B36] Murtaza M. A. Huma N. Sameen A. Murtaza M. S. Mahmood S. Mueen-ud-Din G. (2014). Texture, flavor, and sensory quality of buffalo milk Cheddar cheese as influenced by reducing sodium salt content. *J. Dairy Sci.* 97 6700–6707. 10.3168/jds.2014-8046 25151874

[B37] Murtaza M. A. Huma N. Shabbir M. A. Murtaza M. S. Anees-ur-Rehman M. (2017). Survival of micro-organisms and organic acid profile of probiotic Cheddar cheese from buffalo milk during accelerated ripening. *Int. J. Dairy Technol.* 70 562–571. 10.1111/1471-0307.12406

[B38] Niro S. Succi M. Tremonte P. Sorrentino E. Coppola R. Panfili G. (2017). Evolution of free amino acids during ripening of Caciocavallo cheeses made with different milks. *J. Dairy Sci.* 100 9521–9531. 10.3168/jds.2017-13308 28941817

[B39] Ocak E. Javidipour I. Tuncturk Y. (2015). Volatile compounds of Van Herby cheeses produced with raw and pasteurized milks from different species. *J. Food Sci. Technol.* 52 4315–4323. 10.1007/s13197-014-1458-8 26139896 PMC4486542

[B40] Ozturkoglu-Budak S. Wiebenga A. Bron P. A. de Vries R. P. (2016). Protease and lipase activities of fungal and bacterial strains derived from an artisanal raw ewe’s milk cheese. *Int. J. Food Microbiol.* 237 17–27. 10.1016/j.ijfoodmicro.2016.08.007 27541978

[B41] Panseri S. Chiesa L. M. Zecconi A. Soncini G. De Noni I. (2014). Determination of volatile organic compounds (VOCs) from wrapping films and wrapped PDO Italian cheeses by using HS-SPME and GC/MS. *Molecules* 19 8707–8724. 10.3390/molecules19078707 24968328 PMC6271448

[B42] Park J.-M. Shin J.-A. Lee J. H. Lee K.-T. (2017). Development of a quantitative method for organic acid in wine and beer using high performance liquid chromatography. *Food Sci. Biotechnol.* 26 349–355. 10.1007/s10068-017-0047-9 30263549 PMC6049447

[B43] Paxson H. (2008). Post-pasteurian cultures: the microbiopolitics of raw-milk cheese in the United States. *Cult. Anthropol.* 23 15–47. 10.1111/j.1548-1360.2008.00002.x

[B44] Poveda J. M. Cabezas L. McSweeney P. L. H. (2004). Free amino acid content of Manchego cheese manufactured with different starter cultures and changes throughout ripening. *Food Chem.* 84 213–218. 10.1016/S0308-8146(03)00204-8

[B45] Rehman R.-U. Wang Y. Wang J. Geng W. (2018). Physicochemical analysis of Mozzarella cheese produced and developed by the novel EPS-producing strain Lactobacillus kefiranofaciens ZW3. *Int. J. Dairy Technol.* 71 90–98. 10.1111/1471-0307.12445

[B46] Ryssel M. Johansen P. Al-Soud W. A. Sørensen S. Arneborg N. Jespersen L. (2015). Microbial diversity and dynamics throughout manufacturing and ripening of surface ripened semi-hard Danish Danbo cheeses investigated by culture-independent techniques. *Int. J. Food Microbiol.* 215 124–130. 10.1016/j.ijfoodmicro.2015.09.012 26432602

[B47] Souza C. J. F. Garciarojas E. E. Favarotrindade C. S. (2018). Lactase (β-galactosidase) immobilization by complex formation: impact of biopolymers on enzyme activity. *Food Hydrocoll.* 83 88–96. 10.1016/j.foodhyd.2018.04.044

[B48] Sun Z. Liu W. Bao Q. Zhang J. Hou Q. Kwok L. (2014). Investigation of bacterial and fungal diversity in tarag using high-throughput sequencing. *J. Dairy Sci.* 97 6085–6096. 10.3168/jds.2014-8360 25129502

[B49] van Mastrigt O. Gallegos Tejeda D. Kristensen M. N. Abee T. Smid E. J. (2018). Aroma formation during cheese ripening is best resembled by Lactococcus lactis retentostat cultures. *Microb. Cell Fact.* 17:104. 10.1186/s12934-018-0950-7 29973201 PMC6030761

[B50] Xu M. Jin Z. Lan Y. Rao J. Chen B. (2019). HS-SPME-GC-MS/olfactometry combined with chemometrics to assess the impact of germination on flavor attributes of chickpea, lentil, and yellow pea flours. *Food Chem.* 280 83–95. 10.1016/j.foodchem.2018.12.048 30642511

[B51] Yunita D. Dodd C. E. R. (2018). Microbial community dynamics of a blue-veined raw milk cheese from the United Kingdom. *J. Dairy Sci.* 101 4923–4935. 10.3168/jds.2017-14104 29550118

[B52] Yuvaşen A. Macit E. Dertli E. (2018). Microbial species playing roles for the production of traditional Kasar cheese during pre-maturation period. *LWT* 91 406–413. 10.1016/j.lwt.2018.01.075

[B53] Zheng X. Ge Z. Lin K. Zhang D. Chen Y. Xiao J. (2020). Dynamic changes in bacterial microbiota succession and flavour development during milk fermentation of Kazak artisanal cheese. *Int. Dairy J.* 113:104878 10.1016/j.idairyj.2020.104878

[B54] Zheng X. Liu F. Li K. Shi X. Ni Y. Li B. (2018a). Evaluating the microbial ecology and metabolite profile in Kazak artisanal cheeses from Xinjiang. *China. Food Res. Int.* 111 130–136. 10.1016/j.foodres.2018.05.019 30007669

[B55] Zheng X. Liu F. Shi X. Wang B. Li K. Li B. (2018b). Dynamic correlations between microbiota succession and flavor development involved in the ripening of Kazak artisanal cheese. *Food Res. Int.* 105 733–742. 10.1016/j.foodres.2017.12.007 29433268

[B56] Zhou L. Tang Q. Wasim Iqbal M. Xia Z. Huang F. Li L. (2018). A comparison of milk protein, fat, lactose, total solids and amino acid profiles of three different buffalo breeds in Guangxi. *China. Ital. J. Anim. Sci.* 17 873–878. 10.1080/1828051X.2018.1443288

[B57] Zivkovic M. Cadez N. Uroic K. Miljkovic M. Tolinacki M. Dousova P. (2015). Evaluation of probiotic potential of yeasts isolated from traditional cheeses manufactured in Serbia and Croatia. *J. Intercult. Ethnopharmacol.* 4 12–18. 10.5455/jice.20141128051842 26401378 PMC4566759

