# Supplementary material for: Evaluation of the Effect of Auxiliary Starter Yeasts With Enzyme Activities on Kazak Cheese Quality and Flavor
Source: Front Microbiol. 2020 Dec 16;11:614208. doi: 10.3389/fmicb.2020.614208 (PMC7772356; doi:10.3389/fmicb.2020.614208)
Supplement: Supplementary file 1 [file Data_Sheet_1.pdf]

Supplementary Material

CATALOGUE

Supplementary tables....................2
Table S1. DNA sequence result of yeasts in Kazakh cheese....................2
Table S2. Enzymatic activity of yeasts (U/mL). 3
Table S3. Free amino acid content of four Kazakh cheeses (g/100 g)....................4
Table S4. Organic acid content of four Kazakh cheeses (g/kg) 5
Table S5. Texture analysis of cheeses....................5
Table S6. Odor description and odor activity value (OAV) of aroma compounds in Kazak cheese....................6
Supplementary figures....................7
Figure S1A. Protease hydrolysis diagram of yeasts....................7
Figure S1B. Lipase hydrolysis diagram of yeasts....................8
Figure S1C. β-gaclactosidase hydrolysis diagram of yeasts....................8
Figure S2. PCA analysis of volatile compounds in four Kazakh cheese. 9
Figure S3. Volatile flavor map of four cheeses....................9

Supplementary tables

Table S1. DNA sequence result of yeasts in Kazakh cheese

Strain	Size (bp)	Identification	Similarity (%)	Accession number
A1	568	*Kluyveromyces lactis* strain GG799	99.30	CP021245.1
A2	697	*Kluyveromyces marxianus* strain P2	99.85	KF851351.1
A3	569	*Kluyveromyces lactis* strain CBS 2105	100	CP042459.1
A4	627	*Kluyveromyces marxianus* strain TY17	99.66	FJ972218.1
A5	773	*Torulaspora debrueckii* strain BZL-163	99.48	MN371902.1
A6	646	*Kluyveromyces lactis* strain GG799	99.69	CP021245.1
A7	528	*Lodderomyces elongisporus* strain NX-15	99.24	MN371867.1
A8	544	*Pichia kudriavzevii* strain PK1	99.09	MN648834.1
A9	582	*Pichia kudriavzevii* strain Z2	100	MK310151.1
A10	504	*Candida parapsilosis* strain SLDY-016	99.60	MH748620.1
A11	480	*Pichia kudriavzevii* strain SLDY-035	99.38	MH752055.1
A12	579	*Kluyveromyces marxianus* strain T1	99.31	JX141370.1
A13	561	*Clavispora lusitaniae* strain PC5	99.64	EF063132.1
A14	590	*Lodderomyces elongisporus* strain WY6	99.49	EF643601.1
A15	516	*Candida parapsilosis* strain SLDY-277	99.61	MH782053.1
A16	409	*Pichia fermentans* strain Y1-4	99.03	KF646172.1
A17	549	Clavispora lusitaniae strain TY11	99.45	FJ972214.1
A18	504	*Pichia fermentans* strain NRRL Y-1619	99.21	EF552458.1
A19	461	*Pichia fermentans* strain DF-2	100	KJ638687.1

Table S2. Enzymatic activity of yeasts (U/mL).

Identification	Number	Protease activity	Lipase activity	β-galactosidase activity
Kluyveromyces marxianus	A2	135 ± 17	203 ± 19	217 ± 23
Pichia kudriavzevii	A11	103 ± 10	nd	375 ± 26
Pichia fermentans	A19	111 ± 12	227 ± 23	334 ± 32
Clavispora lusitaniae	A13	86 ± 9	187 ± 18	nd
Lodderomyces elongisporus	A14	94 ± 7	nd	254 ± 20
Candida parapsilosis	A10	121 ± 11	nd	nd
Kluyveromyces marxianus	A4	105 ± 15	147 ± 15	nd
Pichia fermentans	A16	91 ± 17	104 ± 10	nd
Kluyveromyces lactis	A1	82 ± 14	nd	nd
Kluyveromyces marxianus	A12	113 ± 19	nd	243 ± 18
Lodderomyces elongisporus	A7	117 ± 12	200 ± 13	nd
Pichia fermentans	A18	108 ± 15	203 ± 21	nd
Kluyveromyces lactis	A3	114 ± 17	nd	nd
Pichia kudriavzevii	A9	73 ± 8	173 ± 18	nd
Torulaspora debrueckii	A5	85 ± 10	113 ± 13	nd
Pichia kudriavzevii	A8	107 ± 11	127 ± 13	nd

[nd] Means not detected.

[1] Data are mean ± standard deviation of three replicate analyses (n = 3).

Table S3. Free amino acid content of four Kazakh cheeses (g/100 g)

FAA	Control	KmC	PkC	PfC
Asp	0.225 ± 0.003[b]	0.268 ± 0.002[a]	0.225 ± 0.001[b]	0.229 ± 0.002[b]
Thr	0.132 ± 0.001[b]	0.148 ± 0.002[a]	0.129 ± 0.001[c]	0.126 ± 0.002[d]
Ser	0.157 ± 0.002[b]	0.172 ± 0.002[a]	0.147 ± 0.002[c]	0.146 ± 0.003[c]
Glu	0.514 ± 0.003[c]	0.627 ± 0.004[a]	0.503 ± 0.003[d]	0.560 ± 0.004[b]
Gly	0.066 ± 0.001[c]	0.077 ± 0.001[a]	0.068 ± 0.003[b]	0.064 ± 0.002[c]
Ala	0.115 ± 0.001[bc]	0.132 ± 0.001[a]	0.117 ± 0.002[b]	0.113 ± 0.001[c]
Cys	0.002 ± 0.001[a]	0.003 ± 0.001[a]	0.003 ± 0.001[a]	0.002 ± 0.001[a]
Val	0.217 ± 0.003[b]	0.204 ± 0.002[c]	0.228 ± 0.002[a]	0.207 ± 0.001[c]
Met	0.086 ± 0.001[b]	0.096 ± 0.002[a]	0.085 ± 0.001[b]	0.082 ± 0.002[c]
Ile	0.187 ± 0.002[c]	0.211 ± 0.003[a]	0.197 ± 0.001[b]	0.181 ± 0.002[d]
Leu	0.017 ± 0.002[a]	0.016 ± 0.001[a]	0.018 ± 0.003[a]	0.012 ± 0.002[b]
Tyr	0.180 ± 0.002[b]	0.190 ± 0.002[a]	0.161 ± 0.001[c]	0.153 ±0.003[d]
Phe	0.164 ± 0.002[c]	0.187 ± 0.003[a]	0.175 ± 0.002[b]	0.159 ± 0.002[d]
Lys	0.261 ± 0.001[c]	0.291 ± 0.002[a]	0.269 ± 0.002[b]	0.225 ± 0.003[d]
His	0.091 ± 0.001[c]	0.103 ± 0.001[a]	0.095 ± 0.002[b]	0.088 ± 0.002[d]
Arg	0.120 ± 0.002[b]	0.131 ± 0.002[a]	0.118 ± 0.001[b]	0.111 ± 0.002[c]
Pro	0.250 ± 0.001[c]	0.278 ± 0.003[a]	0.259 ± 0.002[b]	0.238 ± 0.003[d]
Total	2.784 ± 0.032[b]	3.134 ± 0.025[a]	2.797 ± 0.027[b]	2.696 ± 0.034[c]

[1] Data are mean ± standard deviation of three replicate analyses (n = 3).

[a-d] Means with different superscripts within the same row are significantly ($P < 0.05$) different.

Table S4. Organic acid content of four Kazakh cheeses (g/kg)

Organic acids	Control	KmC	PkC	PfC
Lactic acid	17.910 ± 1.210^{b}	38.770 ± 3.420^{a}	17.150 ± 1.270^{b}	15.380 ± 1.360^{b}
Malic acid	1.710 ± 0.709^{b}	3.310 ± 0.615^{a}	3.110 ± 0.526^{a}	2.980 ± 0.721^{a}
Citric acid	3.631 ± 0.385^{a}	3.803 ± 0.853^{a}	3.527 ± 0.174^{a}	3.762 ± 0.203^{a}
Tartaric acid	4.864 ± 0.813^{b}	5.318 ± 0.974^{b}	9.492 ± 1.023^{a}	9.241 ± 1.022^{a}
Succinic acid	3.700 ± 0.624^{b}	8.340 ± 1.533^{a}	3.070 ± 0.617^{b}	3.350 ± 1.014^{b}
Total	31.815 ± 3.741^{b}	59.541 ± 7.395^{a}	36.349 ± 3.610^{b}	34.713 ± 4.320^{b}

a Data are mean ± standard deviation of three replicate analyses (n = 3).

[a-d] Means with different superscripts within the same row are significantly ($P < 0.05$) different.

Table S5. Texture analysis of cheeses

Texture	Control	KmC	PkC	PfC
Hardness (N)	2.820 ± 0.150^{a}	2.446 ± 0.120^{b}	1.657 ± 0.110^{c}	1.851 ± 0.130^{c}
Springiness	0.755 ± 0.026^{a}	0.616 ± 0.024^{b}	0.605 ± 0.022^{b}	0.573 ± 0.021^{b}
Cohesiveness	0.514 ± 0.014^{a}	0.452 ± 0.012^{b}	0.510 ± 0.011^{a}	0.501 ± 0.010^{a}
Chewiness (N)	1.450± 0.094^{a}	1.115 ± 0.087^{b}	0.846 ± 0.072^{c}	0.927 ± 0.081^{c}
Gumminess (N)	1.094 ± 0.084^{a}	0.687 ± 0.034^{b}	0.512 ± 0.023^{c}	0.531 ± 0.027^{c}
Chewing resilience	0.253 ± 0.014^{a}	0.197 ± 0.012^{b}	0.207 ± 0.015^{b}	0.204 ± 0.017^{b}

[1] Data are mean ± standard deviation of three replicate analyses (n = 3).

[a-d] Means with different superscripts within the same row are significantly ($P < 0.05$) different.

Table S6. Odor description and odor activity value (OAV) of aroma compounds in Kazak cheese.

Compound	Odor description	Odor threshold [a] (μg/L)	OAV in Control	OAV in KmC	OAV in PkC	OAV in PfC
1-Hexanol	Herbaceous, fruity	5.6	nd	2.57	3.74	1.07
2,3-Butanediol	Fruity, onion	95.1	3.4	42.81	4.38	1.23
Hexanal	Herbaceous	10	nd	3.67	3.03	nd
Nonanal	Citrus-like, oily, rose	8	nd	7.34	1.91	1.74
Ethyl acetate	Fruity, orange	5	124.02	98.79	138.18	53.45
Ethyl butanoate	Fruity, apple	18	nd	9.92	1.70	nd
Isoamyl acetate	Fruity, banana	30	19.04	1.22	19.11	64.37
Ethyl hexanoate	Brandy, orange, sour, fatty	5	7.17	4.71	14.79	3.00
2-Nonanone	Fruity, flower, herbaceous	41	nd	1.34	nd	nd

[1] OAV: odor activity values.

[nd] Means not detected.

[3] The letter indicates odor threshold in water from reference (Gemert, 2011) [a].

Supplementary figures

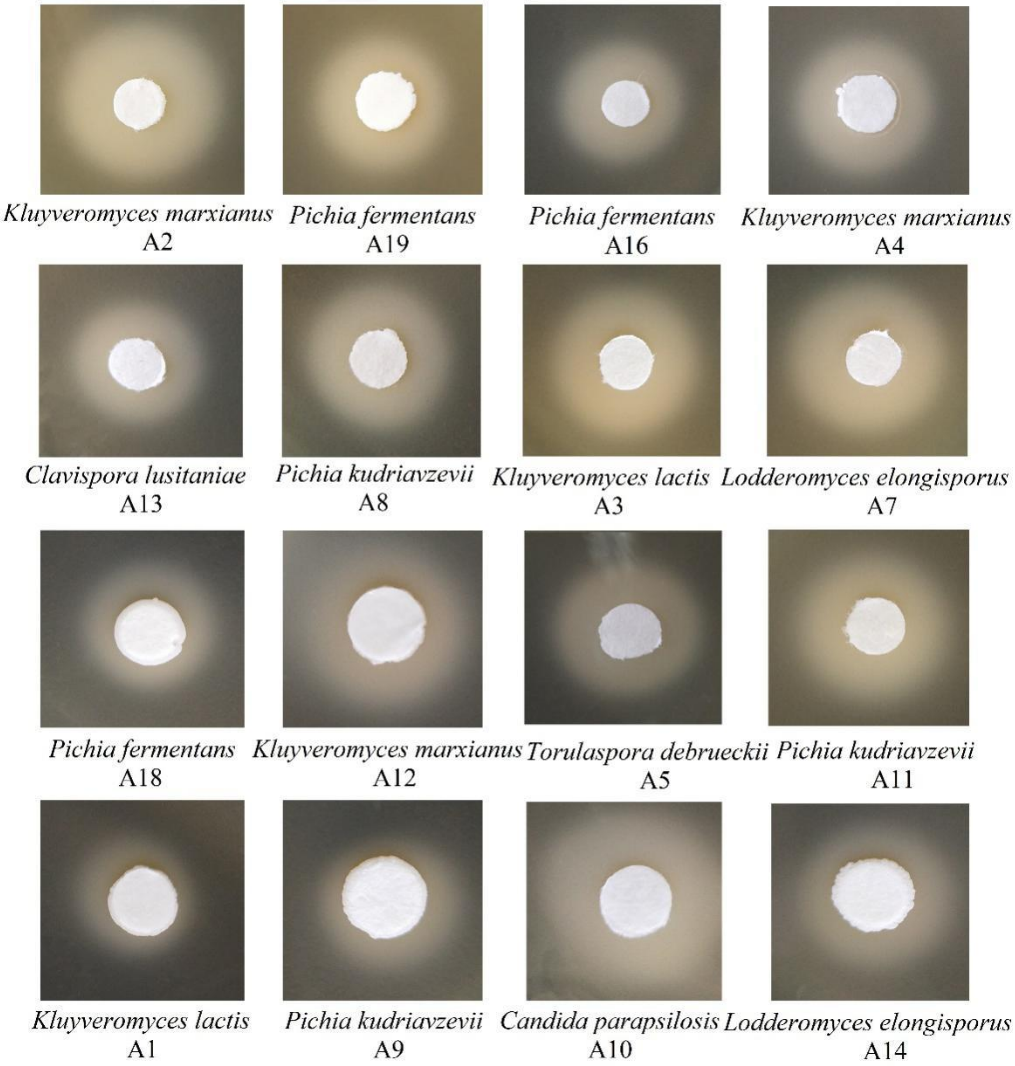

Figure S1A. Protease hydrolysis diagram of yeasts

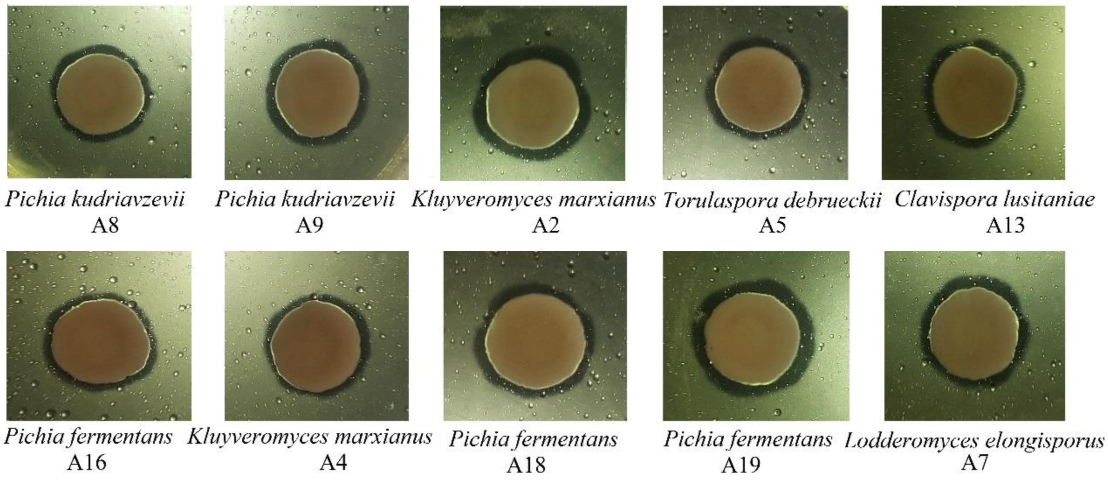

Figure S1B. Lipase hydrolysis diagram of yeasts

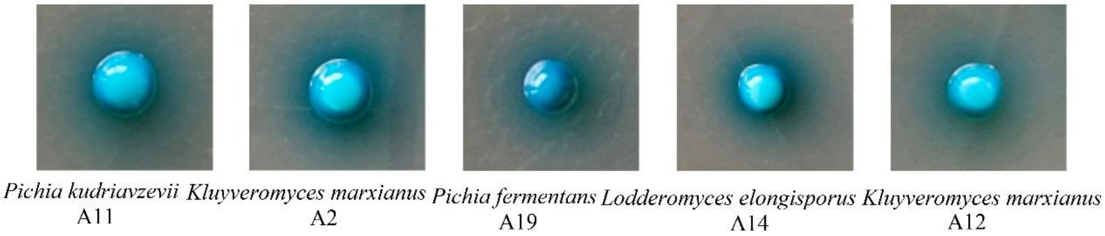

Figure S1C. β -gaclactosidase hydrolysis diagram of yeasts

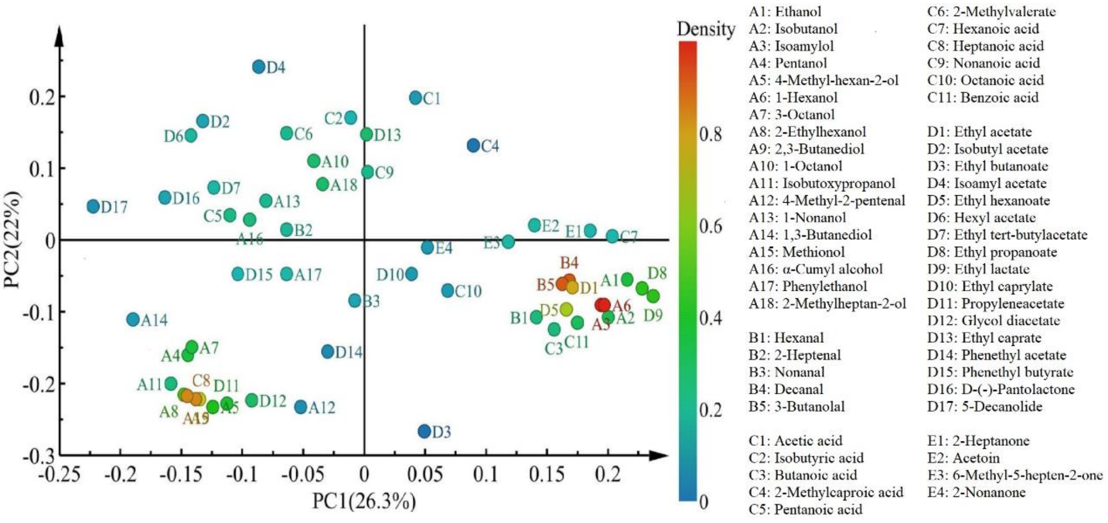

Figure S2. PCA analysis of volatile compounds in four Kazakh cheese.

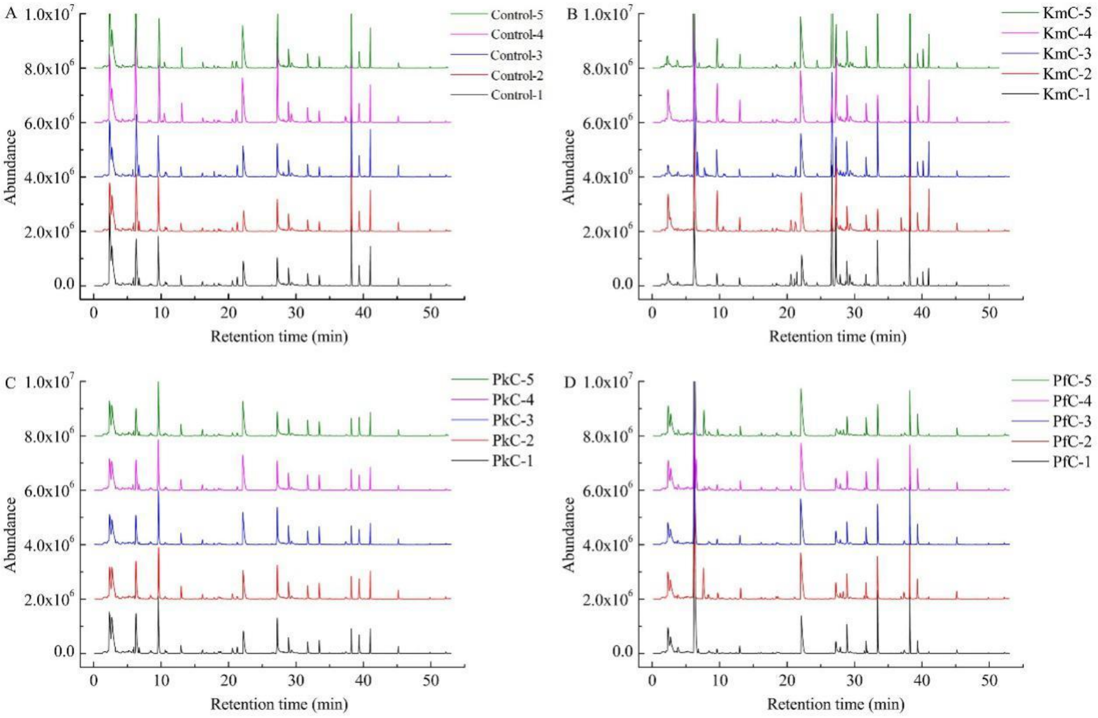

Figure S3. Volatile flavor map of four cheeses
